# Translational tumor control probability modeling for NSCLC: A two‐dimensional maximum‐likelihood framework

**DOI:** 10.1002/mp.70619

**Published:** 2026-07-31

**Authors:** Ryoichi Hinoto, Takeji Sakae, Kenta Takada, Takahisa Eriguchi, Masashi Mizumoto, Atsuya Takeda, Hideyuki Sakurai

**Affiliations:** ^1^ Department of Radiation Oncology Saitama Red Cross Hospital Saitama Saitama Japan; ^2^ Institute of Medicine University of Tsukuba Tsukuba Ibaraki Japan; ^3^ Proton Medical Research Center University of Tsukuba Hospital Tsukuba Ibaraki Japan; ^4^ Graduate School of Radiological Technology Gunma Prefectural College of Health Sciences Maebashi Gunma Japan; ^5^ Department of Radiology Keio University School of Medicine Shinjuku‐ku Tokyo Japan

**Keywords:** errors‐in‐variables, maximum likelihood modeling, non‐small cell lung cancer, radiobiological modeling, tumor control probability

## Abstract

**Background:**

Tumor control probability (TCP) modeling for early‐stage non‐small cell lung cancer (NSCLC) is usually performed by one‐dimensional (1D) fitting that assumes error‐free dose and assigns all uncertainty to the local control (LC) axis. Neglecting dose‐axis uncertainties leads to a classical errors‐in‐variables (EIV) problem that can bias both the dose required for 50% TCP ($\mathrm{TC}D_{50}$) and curve steepness. Furthermore, the mechanistic origins of TCP steepness remain incompletely characterized, limiting the translational link between cellular radiobiology and clinical dose–response.

**Purpose:**

To develop a mechanistic TCP framework that (i) mitigates EIV bias by explicitly modeling uncertainties in both dose and LC, (ii) decomposes TCP steepness into contributions from clonogenic heterogeneity and inter‐patient radiosensitivity, and (iii) validates the translational consistency.

**Methods:**

Cell‐survival data from A549 NSCLC cells were fitted with three radiobiological models—saturable repair and repairability (SRR), universal survival curve (USC), and linear–quadratic (LQ)—using maximum‐likelihood estimation. Clinical TCP modeling used 34 early‐stage NSCLC studies (41 data points, 3‐year LC). Prescriptions were converted to EQD2 using each model's formalism, and TCP curves were fitted by a two‐dimensional (2D) maximum‐likelihood framework. The LC‐axis uncertainty was constructed from binomial sampling uncertainty and an intrinsic scatter, the latter co‐estimated with the effective dose‐axis uncertainty. TCP steepness was measured by the inter‐patient variability $\sigma_{\phi }$ of the TCP exponent and decomposed into clonogenic heterogeneity ($\sigma_{\ln N}$, from reported tumor volumes) and inter‐patient radiosensitivity variability ($\sigma_{E_{2}}$).

**Results:**

In cell‐survival fitting, the SRR and USC models accurately reproduced A549 survival (AIC = −0.49 and 0.21); the LQ model achieved the lowest AIC (−5.17) but only by inflating α/β to 31.1 Gy. In clinical fitting, the SRR, USC, and LQ models yielded ${\mathrm{TCD}}_{50}$ = 57.1, 51.6, and 50.2 Gy EQD2 and ${\mathrm{TCD}}_{95}$ = 81.4, 71.8, and 60.5 Gy. The effective dose‐axis uncertainty (16.6%, 16.9%, and 10.5%) exceeded the mean relative LC‐axis uncertainty (5.0%, 5.0%, and 5.8%), supporting the need for a 2D framework. Variance decomposition revealed that clonogenic heterogeneity contributed only 3%–15% of $\sigma_{\phi }^{2}$, while inter‐patient radiosensitivity dominated (85%–97%); $\sigma_{E_{2}}$ = 0.057–0.120 remained within the CLARIFi‐derived inter‐cell‐line $\sigma_{E_{2}}$ bound of 0.255 (across 41 NSCLC cell lines). Fitted $N_{0}$ for the SRR and USC models implied mean tumor diameters of 2.6 and 3.2 cm, both falling within approximately one standard deviation of the clinical reference 2.7±0.6 cm; the LQ‐model substantially overestimated the diameter (6.0 cm).

**Conclusions:**

The proposed 2D maximum‐likelihood framework mitigates EIV bias and links clinical TCP to mechanistic cell‐survival models. In early‐stage NSCLC, inter‐patient radiosensitivity dominates TCP steepness while clonogenic cell‐number variability contributes negligibly. This framework provides a robust basis for future TCP modeling and dose‐prescription studies.

## 1 INTRODUCTION

Tumor control probability (TCP) modeling seeks to translate radiobiological principles into clinical predictions, yet commonly used approaches retain key methodological gaps. Most clinical studies construct TCP curves through one‐dimensional (1D) optimization that treats the delivered dose as error‐free and assigns all statistical uncertainty to the local control (LC) axis.^1^, ^2^, ^3^, ^4^ In reality, the dose delivered to the tumor is subject to inter‐cohort and inter‐patient variability arising from (i) treatment delivery, (ii) dose‐calculation algorithms, and (iii) contouring accuracy, collectively introducing uncertainty along the dose‐axis. Neglecting these sources yields a classical *errors‐in‐variables* (EIV) problem and can bias both the position and the steepness of the fitted TCP curve. Santiago et al.^2^ recognized this limitation, reporting pronounced flattening in their TCP modeling; yet they did not provide a concrete means of incorporating dose‐axis uncertainty.

The deeper, unresolved question is *why* TCP steepness varies biologically. Bentzen and Tucker^5^ noted that patient‐to‐patient heterogeneity—both biological and geometrical—underlies the shape of clinical dose–response curves. Within the Poisson TCP framework, the exponent combines a term related to the total number of clonogenic cells and a dose‐dependent term describing the effective cell kill. Variability in these quantities across patients is thought to drive the observed TCP steepness.^6^

Several mechanistic TCP formalisms have been proposed to represent inter‐patient variation. Webb and Nahum^6^ incorporated spatial heterogeneity in clonogenic cell density and dose, demonstrating that non‐uniform cell distributions can broaden the population dose–response. Keall and Webb^7^ extended this by modeling inter‐patient variability in tumor radiosensitivity, treating it as a distributed quantity rather than a single fixed value. These studies established that biological heterogeneity—whether in clonogenic burden or radiosensitivity—fundamentally shapes TCP curves. However, the relative contribution of clonogenic cell variability versus dose–response variability to the observed clinical TCP slope has not been quantified systematically.

Here, we address these methodological and mechanistic gaps through two innovations. First, we implement a two‐dimensional (2D) maximum‐likelihood framework that estimates uncertainty in *both* dose and LC, thereby mitigating the classical EIV bias and providing statistically robust estimates. Second, we introduce a mechanistic formulation in which TCP steepness is governed by heterogeneity in clonogenic cell burden and in the effective dose‐dependent cell kill term, yielding a biologically grounded framework that decomposes observed TCP slopes into contributions from these two sources.

## 2 METHODS

### 2.1 Theoretical framework

#### 2.1.1 TCP model formulation

TCP represents a quantitative measure of the likelihood of achieving complete tumor eradication following radiotherapy. The classical formulation of TCP is based on Poisson statistics, where the probability of tumor control is equivalent to the probability that no clonogenic cells survive the treatment.^8^, ^9^

The mathematical expression for TCP is given by:

(1) $$\mathrm{TCP}={\left(1-S\right)}^{N}=\exp\left(N\ln(1-S)\right)$$

where S represents the surviving fraction of clonogenic cells after irradiation, and N denotes the initial number of clonogenic cells in the tumor before treatment. For clinical scenarios where the surviving fraction is sufficiently small (S≪1), which is typically the case in effective radiotherapy regimens,^10^ the logarithmic term can be approximated as ln(1−S)≈−S, yielding:

(2) $$\mathrm{TCP}\approx \exp\left(-NS\right)$$

The TCP model incorporates radiobiological models through the surviving fraction S calculated using appropriate equivalent dose in 2 Gy fractions (EQD2) formulations. For computational efficiency, the TCP model is expressed as:

(3) $$\mathrm{TCP}=\exp\left(-N\cdot \exp\left(-\frac{E_{2}}{2}\cdot \mathrm{EQD}2\right)\right),$$

where $E_{2}=-\ln\left(S\left(2\right)\right)$ is the effect per 2 Gy fraction.

The critical parameter ${\mathrm{TCD}}_{50}$, defined as the EQD2 at which TCP = 0.5, is related to the initial clonogenic cell number:

(4) $${\mathrm{TCD}}_{50}=\frac{2}{E_{2}}\cdot \left(\ln\left(N\right)-\ln\left(\ln\left(2\right)\right)\right)$$

#### 2.1.2 A steepness framework for TCP with explicit uncertainties

Dose–response steepness is conventionally summarized by the normalized gradient at the 50% point:^5^, ^11^

(5) $$\gamma_{50}\;\equiv \;{\mathrm{TCD}}_{50}\;\frac{d\;\mathrm{TCP}}{d\;\mathrm{EQD}2}{|}_{\mathrm{EQD}2={\mathrm{TCD}}_{50}}.$$

This quantity is model‐agnostic and comparable across sigmoid forms. From Equation (3), the 50% condition $N\;\exp\;\left(-\frac{E_{2}}{2}\;{\mathrm{TCD}}_{50}\right)=\ln2$ leads directly to the mechanistic link

(6) $$\gamma_{50}\;=\;\frac{E_{2}\;{\mathrm{TCD}}_{50}\;\ln2}{4},$$

connecting Bentzen's phenomenological steepness to the radiobiological parameter $E_{2}$.

From Equation (3), we define the log–log transformed variable

(7) $$\phi \left(\mathrm{EQD}2;N,E_{2}\right)\;\equiv \;\ln N\;-\;\frac{E_{2}}{2}\;\mathrm{EQD}2,$$

so that ln(−lnTCP)=ϕ. Inter‐patient heterogeneity is then quantified by the variance at ${\mathrm{TCD}}_{50}$

(8) $$\sigma_{\phi }^{2}\;\equiv \;\mathrm{Var}\;\left(\phi \right){|}_{\mathrm{EQD}2={\mathrm{TCD}}_{50}}.$$

When ϕ varies across patients with variance $\sigma_{\phi }^{2}$, Gaussian convolution of Equation (3) under a Gaussian‐sigmoid approximation reduces the slope by a correction factor

(9) $$m\;=\;\sqrt{\;1+2\pi {\left(\frac{\ln2}{2}\right)}^{\;2}\;\sigma_{\phi }^{2}\;},\;m\geq 1,$$

yielding the effective gradient

(10) $$\gamma_{50}^{\text{(eff)}}\;=\;\frac{\gamma_{50}}{m}.$$

Embedding this slope correction into the Poisson TCP framework while preserving ${\mathrm{TCD}}_{50}$ gives,

(11) $$\begin{array}{cc} & \mathrm{TCP}(\mathrm{EQD}2)\\ & =\exp\;\left[-N\exp\left(-\frac{E_{2}}{2}\left(\frac{\mathrm{EQD}2-{\mathrm{TCD}}_{50}}{m}+{\mathrm{TCD}}_{50}\right)\right)\right], \end{array}$$

where m=1 recovers the homogeneous case and m>1 represents heterogeneity‐driven slope relaxation. The resulting TCP curve provides a slope‐corrected approximation to the population‐averaged response.

#### 2.1.3 Representative clinical dose

To use the TCP framework of Equations (3) and (11), each clinical regimen requires a single representative dose. This choice is nontrivial for stereotactic body radiotherapy (SBRT), where peripheral‐isodose prescription produces central hot spots within the target.

We based this choice on the survival‐based form of the equivalent uniform dose (EUD), originally introduced by Niemierko^12^ as the uniform dose that yields the same volume‐averaged clonogenic surviving fraction as the inhomogeneous dose distribution. This definition preserves the Poisson TCP of Equation (3) and is to be distinguished from the later generalized EUD.^13^ In the SBRT dose range, the volume‐averaged surviving fraction is governed by the low‐dose tail of the target dose distribution rather than by central hot spots.^14^ We therefore used the PTV peripheral dose as a practical surrogate for this low‐dose region.

#### 2.1.4 Tumor repopulation correction

The TCP model in Equation (3) does not include an explicit tumor repopulation term. We therefore corrected each clinical EQD2 for clonogenic regrowth during treatment before fitting. Based on the standard accelerated‐repopulation formulation,^15^, ^16^ the repopulation‐corrected EQD2 is given by

(12) $$\mathrm{EQD}2_{\mathrm{rep}}\;=\;\mathrm{EQD}2-\frac{2}{E_{2}}\;\mu_{g}\;\max\left(T-T_{k},\;0\right),$$

where $\mu_{g}=\ln2/T_{TD}$ is the clonogenic repopulation rate; T=1.4(n−1) days is the overall treatment time for the standard 5‐fraction‐per‐week schedule; and $T_{k}$ is the kick‐off time for accelerated repopulation. We adopted $T_{k}=21$ days, the lower bound of the range $T_{k}=21$–32 days recommended for NSCLC.^16^, ^17^ The tumor doubling time $T_{TD}=150$ days was obtained by weighting the NSCLC subtype‐specific doubling times reported by Mizuno et al.^18^ by the contemporary global NSCLC histology distribution,^19^ giving $\mu_{g}=4.62\times 10^{-3}$ ${\mathrm{day}}^{-1}$. SBRT regimens with $T\leq T_{k}$ receive no correction. Throughout this manuscript, EQD2 denotes this repopulation‐corrected value $\mathrm{EQD}2_{\mathrm{rep}}$.

### 2.2 Radiobiological models

To implement our TCP theoretical framework, we employed three distinct radiobiological models that bridge cellular and clinical data: the SRR‐model (Saturable Repair and Repairability model),^20^ the USC‐model (Universal Survival Curve model),^21^ and the LQ‐model (Linear‐Quadratic model).^22^ These models were selected for their complementary approaches to modeling radiation response across different dose ranges, enabling validation of our framework's generalizability across diverse radiobiological assumptions.

#### 2.2.1 Saturable repair and repairability model

The SRR‐model incorporates the concept of saturable repairability of radiation damage, accounting for the finite repair capacity that can be overwhelmed by excessive damage.

The mathematical formulation of the SRR‐model for cell survival S(d) after irradiation with dose d is given by:

(13) $$S\left(d\right)=\exp\left(-K\left(d\right)+fR\left(1-\exp\left(-\frac{K(d)}{R}\right)\right)\right)$$

where K(d) represents the cumulative damage function defined as:

(14) $$K\left(d\right)=ad-\ln\left(2-c_{1}-\left(1-c_{1}\right)\exp\left(-ad\right)\right)$$

The key parameters include: a (linear efficiency of radiation damage), R (cell‐specific repairability capacity), $c_{1}$ (probability of affected damage), and f (proportion of repairable damage). For this analysis, $c_{1}$ and f were fixed at 0.02 and 0.999, respectively, representing typical values for low linear energy transfer radiation.

The EQD2 for the SRR‐model is calculated as:

(15) $${\text{EQD2}}_{\text{SRR}}=2\times n\times \frac{K\left(d\right)-fR\left(1-\exp\left(-\frac{K(d)}{R}\right)\right)}{K\left(2\right)-fR\left(1-\exp\left(-\frac{K(2)}{R}\right)\right)}$$

where n is the number of fractions and d is the dose per fraction in Gy.

#### 2.2.2 Universal survival curve model

The USC‐model combines the LQ‐model for low doses with a linear model for high doses, incorporating a transition dose ($d_{t}$) at which the survival curve transitions from curved to linear response.

The mathematical formulation of the USC‐model for cell survival S(d) after irradiation with dose d is given by:

(16) $$S\left(d\right)=\left\{\begin{array}{cc} \exp(-\alpha d-\beta d^{2}) & \text{if}\;d\leq d_{t}\\ \exp(-\alpha d_{t}-\beta d_{t}^{2}-\left(\alpha +2\beta d_{t}\right)\left(d-d_{t}\right)) & \text{if}\;d>d_{t} \end{array}\right.$$

where α and β are the linear and quadratic coefficients, and $d_{t}$ is the transition dose. The EQD2 for the USC‐model is calculated as:

(17) $${\text{EQD2}}_{\text{USC}}=\left\{\begin{array}{cc} nd\frac{\alpha /\beta +d}{\alpha /\beta +2} & \text{if}\;d\leq d_{t}\\ n\left[d_{t}\frac{\alpha /\beta +d_{t}}{\alpha /\beta +2}+\left(d-d_{t}\right)\frac{\alpha /\beta +2d_{t}}{\alpha /\beta +2}\right] & \text{if}\;d>d_{t} \end{array}\right.$$

where n is the number of fractions and d is the dose per fraction in Gy.

#### 2.2.3 Linear‐quadratic model

The LQ‐model corresponds to the low‐dose region of the USC‐model, specifically when $d\leq d_{t}$. The cell survival function S(d) and EQD2 calculation for the LQ‐model are identical to the USC‐model formulations in the low‐dose regime:

(18) $$S\left(d\right)=\exp\left(-\alpha d-\beta d^{2}\right)$$

(19) $${\text{EQD2}}_{\text{LQ}}=nd\frac{\alpha /\beta +d}{\alpha /\beta +2}$$

### 2.3 Data collection

#### 2.3.1 Cell survival data

We utilized published cell survival data from Saga et al.^23^ using the A549 non‐small cell lung cancer (NSCLC) cell line, which included dose–response measurements across 0–15 Gy. A549 was selected as representative of the predominant NSCLC histological subtype globally.^19^ Importantly, A549 also represents the central tendency of the surviving fraction at 2 Gy (${\mathrm{SF}}_{2}$; numerically equivalent to S(2) in our model notation) across NSCLC cell lines, with its median value (${\mathrm{mSF}}_{2}$) closely matching the NSCLC‐wide median in the CLARIFi database.^24^ This central‐tendency property is essential because the TCP curve constructed from multi‐institutional clinical data represents the population‐averaged response.

#### 2.3.2 Clinical data

A systematic literature search was conducted following PRISMA guidelines.^25^ We searched PubMed for English‐language publications (January 2000–December 2021). Studies were included if they met: (1) median follow‐up ≥15 months; (2) sample size ≥25 patients; (3) early‐stage NSCLC; (4) photon radiotherapy alone; (5) reported 3‐year LC rates; and (6) reporting of the PTV peripheral dose, or of another dose metric that allowed conversion to an equivalent PTV peripheral dose. The 3‐year LC endpoint was adopted in accordance with Sanuki et al.,^26^ who demonstrated its correlation with overall survival.

Following the selection criteria, 34 studies yielding 41 data points were included. The detailed search strategy and complete list of included studies are provided in the Supplementary Materials.

### 2.4 Optimization framework for radiobiological model parameters and TCP curve fitting

The framework followed a two‐phase approach: first, optimization of radiobiological model parameters from in vitro cell survival data; second, TCP curve fitting to clinical outcomes with the cell model parameters held fixed.

#### 2.4.1 Model parameter optimization

For the first phase, we optimized radiobiological model parameters exclusively using in vitro cell survival data obtained from the A549 cell line. To avoid potential bias from individual uncertainty estimates, we adopted a global proportional uncertainty approach. The objective function was formulated in log‐survival space:

(20) $$F_{\text{cell}}=\sum_{i}\frac{{\left(\ln S_{o,i}-\ln S_{p,i}\right)}^{2}}{u_{\text{SF}}^{2}}$$

where $S_{o,i}$ and $S_{p,i}$ represent the observed and predicted cell survival fractions, respectively, and $u_{\text{SF}}$ is a global uncertainty scale in log‐survival space, co‐estimated with the model parameters by maximizing the corresponding Gaussian log‐likelihood:

(21) $$\ln L=-\frac{1}{2}\sum_{i}\left[\ln\left(2\pi \right)+2\ln u_{\text{SF}}+\frac{{\left(\ln S_{o,i}-\ln S_{p,i}\right)}^{2}}{u_{\text{SF}}^{2}}\right]$$

This formulation assigns the same proportional uncertainty to all dose points.

For the SRR‐model, optimization involved simultaneously determining the linear efficiency (a), cell‐specific repairability (R), and $u_{\text{SF}}$. For the LQ‐model, α, β, and $u_{\text{SF}}$ were optimized simultaneously. The USC‐model required a hierarchical optimization strategy to resolve the structural coupling between α, β, and $d_{t}$. Because $d_{t}$ is unknown prior to fitting, we adopted an iterative approach. Prior reports suggest $d_{t}$ in the range of 5–6 Gy for this cell line;^23^, ^27^ we therefore initially determined α and β using data points with doses ≤6 Gy, noting that the validity of this threshold is governed by the α/β ratio rather than a fixed physical dose .^22^, ^28^ The α and β parameters were then fixed while optimizing $d_{t}$ and $u_{\text{SF}}$ across the complete dataset.

Parameter uncertainties for a, α, and β were quantified through the Fisher information matrix. For the repair parameter R of the SRR‐model and the transition dose $d_{t}$ of the USC‐model, we report profile likelihood confidence intervals: R is subject to a physical non‐negativity constraint (R≥0), and $d_{t}$ is structurally coupled with α and β through the USC‐model formulation. For such parameters, Fisher‐based symmetric approximations can be inadequate when the likelihood surface is bounded or asymmetric, motivating the use of profile likelihood. The profile likelihood for a parameter of interest $\theta_{j}$ is obtained by fixing $\theta_{j}$ at a grid of values and re‐optimizing the remaining parameters at each grid point; the 95% confidence interval is defined by the set of $\theta_{j}$ values satisfying

(22) $$2\left[\ln{\hat{L}}_{\max}-\ln{\hat{L}}_{\text{profile}}\left(\theta_{j}\right)\right]<\chi_{1,\;0.95}^{2}$$

where ${\hat{L}}_{\max}$ is the global maximum likelihood and $\chi_{1,\;0.95}^{2}=3.841$ is the 95th percentile of the chi‐squared distribution with one degree of freedom. For $d_{t}$, consistent with the hierarchical fitting strategy described above, the profile likelihood is evaluated with α and β held at their low‐dose estimates; only $u_{\text{SF}}$ is re‐optimized at each grid value of $d_{t}$.

All optimizations utilized a differential evolution algorithm^29^ with standardized hyperparameters: population size of 50 individuals, 500 maximum iterations, adaptive mutation factor ranging from 0.5 to 1.0, and crossover probability of 0.7.

#### 2.4.2 TCP curve fitting

For each radiobiological model, cohort regimens were converted to a model‐specific EQD2 with the repopulation correction of Equation (12). The TCP curve was then fitted to the resulting $\left(\mathrm{EQD}2_{o,i},\;{\mathrm{LC}}_{o,i}\right)$ pairs by maximum likelihood under a 2D formulation. The dose‐ and LC‐axis errors were modeled as independent Gaussians with standard deviations proportional to the observed magnitudes:

(23) $$\sigma_{\mathrm{EQD}2,i}=u_{\mathrm{EQD}2}\;\mathrm{EQD}2_{o,i},$$

(24) $$\sigma_{\mathrm{LC},i}^{2}=\sigma_{\mathrm{Jeff},i}^{2}+\max\;\left(0,\;\sigma_{\mathrm{int}}^{2}-{\left({\mathrm{TCP}}^{\prime }\left(\mathrm{EQD}2_{o,i}\right)\;\sigma_{\mathrm{EQD}2,i}\right)}^{2}\right).$$

where $\mathrm{EQD}2_{o,i}$ and ${\mathrm{LC}}_{o,i}$ are the observed EQD2 and LC values, respectively, and the global scale $u_{\mathrm{EQD}2}$ captures the overall proportional uncertainty in EQD2 across heterogeneous data sources. The LC‐axis variance $\sigma_{\mathrm{LC},i}^{2}$ has two components. The first is the Jeffreys posterior variance of the binomial proportion for the cohort LC rate; this is the irreducible sampling component and is tabulated per cohort in Table S‐1. The second is the intrinsic LC‐axis scatter beyond binomial sampling, reflecting inter‐institutional variability, summarized by $\sigma_{\mathrm{int}}$ with literature‐supported range.^30^, ^31^, ^32^ Because the literature studies aggregated in $\sigma_{\mathrm{int}}$ do not separately resolve LC‐axis assessment noise from dose‐related scatter, $\sigma_{\mathrm{int}}$ reflects the total observable LC scatter and already implicitly includes the slope‐projected component of dose‐axis uncertainty. To avoid double‐counting this budget on both axes, the $\sigma_{\mathrm{int}}^{2}$ budget is allocated dynamically through the curve slope, where ${\mathrm{TCP}}^{\prime }\left(\mathrm{EQD}2\right)\equiv d\mathrm{TCP}/d\mathrm{EQD}2$ denotes the local slope of the TCP curve. Under this allocation, the total LC‐axis variance budget remains $\sigma_{\mathrm{Jeff}}^{2}+\sigma_{\mathrm{int}}^{2}$ in the plateau and transition regimes; only the allocation of the $\sigma_{\mathrm{int}}^{2}$ budget between the LC‐axis and slope‐projected dose‐axis terms shifts with curve slope. We treat $\sigma_{\mathrm{int}}$ as the fourth free parameter, bounded by the literature‐supported range [0,0.12] — the lower bound corresponding to full absorption into the dose‐axis uncertainty under the joint‐floor formulation, the upper bound to the literature maximum.^30^, ^31^, ^32^

Given a TCP model LC=TCP(EQD2;θ) parameterized by θ (including the clonogen number N that sets the horizontal position and the slope correction factor m that controls the curve steepness), each observation $\left(\mathrm{EQD}2_{o,i},{\mathrm{LC}}_{o,i}\right)$ is projected onto the model curve by minimizing the weighted Mahalanobis orthogonal distance:

(25) $$\begin{array}{cc} \mathrm{EQD}2_{i}^{*}\left(\theta \right) & =\arg\min_{\mathrm{EQD}2}\frac{{\left(\mathrm{EQD}2_{o,i}-\mathrm{EQD}2\right)}^{2}}{\sigma_{\mathrm{EQD}2,i}^{2}}\\ & \;+\frac{{\left({\mathrm{LC}}_{o,i}-\mathrm{TCP}\left(\mathrm{EQD}2;\theta \right)\right)}^{2}}{\sigma_{\mathrm{LC},i}^{2}}, \end{array}$$

(26) $${\mathrm{LC}}_{i}^{*}\left(\theta \right)=\mathrm{TCP}\left(\mathrm{EQD}2_{i}^{*};\theta \right)$$

with $\mathrm{EQD}2_{i}^{*}\left(\theta \right)$ obtained numerically for each i and θ.

The likelihood is

(27) $$\begin{array}{cc} & L\left(\theta ,\;u_{\mathrm{EQD}2},\;\sigma_{\mathrm{int}}\right)=\prod_{i=1}^{n}\frac{1}{2\pi \;\sigma_{\mathrm{EQD}2,i}\sigma_{\mathrm{LC},i}}\\ & \;\times \exp\;\left(-\frac{{\left(\mathrm{EQD}2_{o,i}-\mathrm{EQD}2_{i}^{*}\right)}^{2}}{2\sigma_{\mathrm{EQD}2,i}^{2}}-\frac{{\left({\mathrm{LC}}_{o,i}-{\mathrm{LC}}_{i}^{*}\right)}^{2}}{2\sigma_{\mathrm{LC},i}^{2}}\right) \end{array}$$

and its maximized value is

(28) $$\hat{L}=L\;\left(\hat{\theta },\;{\hat{u}}_{\mathrm{EQD}2},\;{\hat{\sigma }}_{\mathrm{int}}\right).$$

We then minimize the Akaike Information Criterion (AIC),^33^

(29) $$\mathrm{AIC}=2k-2\ln\hat{L},$$

where k is the number of free parameters estimated (here k=4: N, m, $u_{\mathrm{EQD}2}$, $\sigma_{\mathrm{int}}$). A step‐by‐step derivation of the bivariate likelihood, its log form, and the corresponding AIC formula is provided in Appendix A.

The 2D likelihood was minimized by the same differential evolution algorithm^29^ used for cell‐survival fitting, with population size 15, 300 maximum iterations, and a dithered mutation factor ranging from 0.5 to 1.5. To reduce sensitivity to stochastic initialization, each fit was repeated with seven independent seeds, and the best objective value was retained. Parameter bounds were $N\in [10,\;10^{15}]$, m∈[1,20], $u_{\mathrm{EQD}2}\in \left[0.10,\;1.00\right]$, and $\sigma_{\mathrm{int}}\in \left[0,\;0.12\right]$.

### 2.5 Goodness‐of‐fit evaluation

To compare the three models, we used AIC as the primary metric. Lower AIC values indicate a better balance between model fit and complexity. Models with ΔAIC ≤2 are generally regarded as having substantial support relative to the best‐fitting model, those with 4≤ΔAIC ≤7 have considerably less support, and those with ΔAIC >10 have essentially no support.^34^

#### 2.5.1 AIC for cell survival data

For the in vitro cell‐survival datasets, we reused the Gaussian likelihood defined in Section 2.4.1, where residuals were computed in log‐survival space; the global scale $u_{\text{SF}}$ was co‐estimated with the cell‐survival model parameters. The maximized likelihood for cell‐survival data is ${\hat{L}}_{\text{cell}}=L\left(\hat{\theta },{\hat{u}}_{\text{SF}}\right)$. Model comparison was based on

(30) $${\mathrm{AIC}}_{\text{cell}}=2k-2\ln\left({\hat{L}}_{\text{cell}}\right),$$

where k is the number of free parameters in the survival model.

#### 2.5.2 AIC for clinical data

For clinical EQD2–LC pairs, we reused the bivariate Gaussian likelihood with the joint‐floor LC‐axis variance defined in Section 2.4.2; the dose‐axis scale $u_{\mathrm{EQD}2}$ and the intrinsic LC scatter $\sigma_{\mathrm{int}}$ were co‐estimated with the TCP‐curve parameters N and m. The maximized likelihood for clinical data is

(31) $${\hat{L}}_{\text{clinical}}=L\;\left(\hat{\theta },\;{\hat{u}}_{\mathrm{EQD}2},\;{\hat{\sigma }}_{\mathrm{int}}\right),$$

where L is the bivariate Gaussian likelihood defined in Equation (28). Model comparison was based on

(32) $${\mathrm{AIC}}_{\text{clinical}}=2k-2\ln\left({\hat{L}}_{\text{clinical}}\right),$$

with k=4 (N, m, $u_{\mathrm{EQD}2}$, $\sigma_{\mathrm{int}}$).

### 2.6 Conditional variance decomposition at ${\mathrm{TCD}}_{50}$

The slope correction factor m (Equation 9) encodes the inter‐patient TCP exponent variance via,

(33) $$\sigma_{\phi }^{2}=\frac{m^{2}-1}{2\pi {\left(\frac{\ln2}{2}\right)}^{2}}.$$

Since $\sigma_{\phi }^{2}$ is the *conditional* variance of ϕ at $\mathrm{EQD}2={\mathrm{TCD}}_{50}$ (Equation 8), the reference dose acts as a deterministic scalar in the linear expression $\phi =\ln N-(E_{2}/2)\;\mathrm{EQD}2$, yielding

(34) $$\begin{array}{cc} \sigma_{\phi }^{2} & =\sigma_{\ln N}^{2}+{\left(\frac{{\mathrm{TCD}}_{50}}{2}\right)}^{\;2}\sigma_{E_{2}}^{2}\\ & \;-{\mathrm{TCD}}_{50}\;\mathrm{Cov}\left(\ln N,E_{2}\right)+\sigma_{\mathrm{meta}}^{2}, \end{array}$$

where $\sigma_{\ln N}^{2}$ and $\sigma_{E_{2}}^{2}$ are inter‐patient variances in clonogen number (lnN assumed to follow approximately a normal distribution^35^) and intrinsic radiosensitivity, $\mathrm{Cov}(\ln N,E_{2})$ is any inter‐patient correlation between them, and $\sigma_{\mathrm{meta}}^{2}$ absorbs residual variance not captured by the linear $\left(\ln N,E_{2}\right)$ decomposition.

As a minimal backward‐inference model we set $\mathrm{Cov}(\ln N,E_{2})=0$ and $\sigma_{\mathrm{meta}}=0$. These are methodological choices that define the residual to vanish by construction rather than claims that biological covariance or unmodeled variance are absent; they yield

(35) $$\sigma_{E_{2}}=\frac{2}{{\mathrm{TCD}}_{50}}\sqrt{\sigma_{\phi }^{2}-\sigma_{\ln N}^{2}}.$$

An inferred $\sigma_{E_{2}}$ within an externally constrained upper bound indicates self‐consistency of the minimal model; exceeding the bound would imply nonzero Cov or $\sigma_{\mathrm{meta}}$. We take ${\mathrm{CV}}_{{\mathrm{SF}}_{2}}=0.255$ from the inter‐cell‐line distribution of ${\mathrm{mSF}}_{2}$ in the CLARIFi database;^24^ via the linearization $E_{2}=-\ln{\mathrm{SF}}_{2}$ ($\sigma_{E_{2}}\approx {\mathrm{CV}}_{{\mathrm{SF}}_{2}}$), this yields $\sigma_{E_{2}}≲0.255$ as the upper bound.

The clonogenic cell variability $\sigma_{\ln N}$ was estimated independently of the TCP model using tumor volume data reported in the clinical studies (Table S‐1). Specifically, we collected the representative tumor volume (mean or median) from each study, computed lnV for each, and calculated the standard deviation of these lnV values across studies. Assuming a constant clonogenic cell density ρ (so that N=ρV and lnN=lnρ+lnV), this yields $\sigma_{\ln N}=\sigma_{\ln V}$. Because $\sigma_{\ln N}$ characterizes between‐study heterogeneity in tumor size, each study's representative volume was given equal weight.

To validate the translational framework, we translated the fitted $N_{0}$ and $\sigma_{\ln N}$ into tumor diameter distributions, assuming log‐normal N and spherical tumor geometry with $\rho =10^{7}$ clonogens/${\mathrm{cm}}^{3}$.^6^, ^23^

## 3 RESULTS

### 3.1 Cell survival model parameters

**FIGURE 1:**
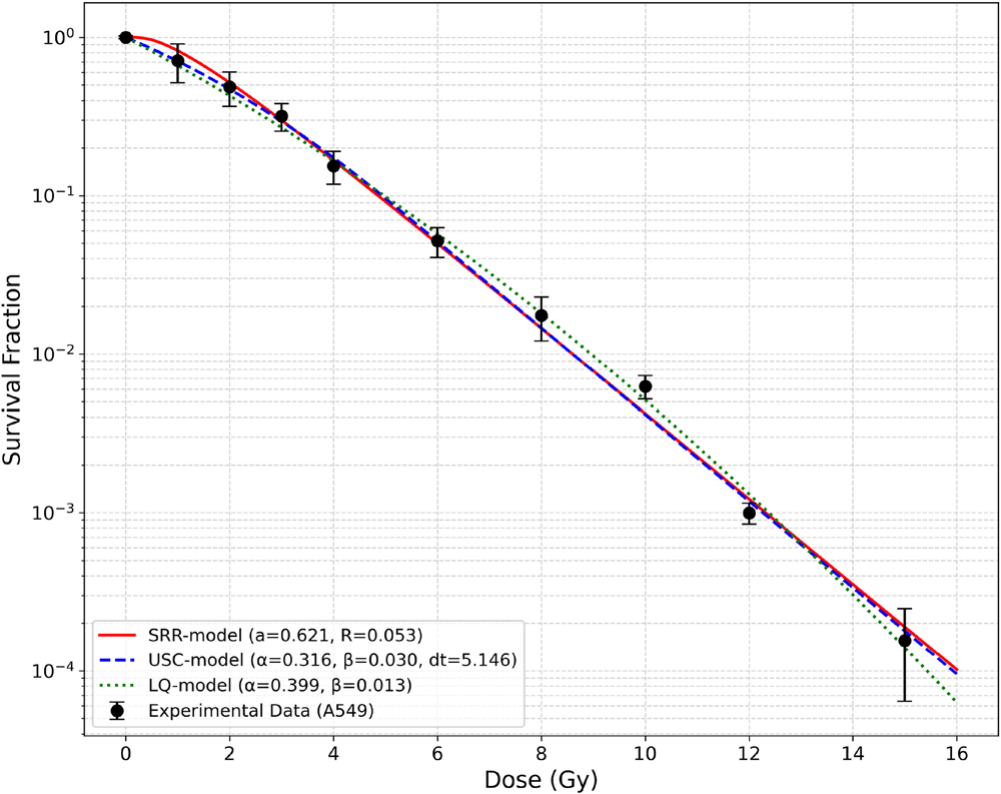
Cell survival curves for A549 cells fitted with SRR‐model (red solid line), USC‐model (blue dashed line), and LQ‐model (green dotted line). Experimental data from Saga et al.^23^ with error bars representing reported standard deviations. All three models were fitted in log‐survival space using a global proportional uncertainty $u_{\text{SF}}$.

All three radiobiological models were fitted to A549 cell survival data across the 0–15 Gy dose range (Figure 1). The SRR‐model yielded parameters a=0.621±0.014 ${\mathrm{Gy}}^{-1}$ and R=0.053 (95% profile likelihood CI: [0.000, 0.330]; Appendix B). The USC‐model parameters were α=0.316±0.070 ${\mathrm{Gy}}^{-1}$, β=0.030±0.014 ${\mathrm{Gy}}^{-2}$ (α/β=10.4 Gy), and $d_{t}=5.146$ Gy (95% profile likelihood CI: [4.80, 5.53] Gy; Appendix B). The LQ‐model yielded α=0.399±0.020 ${\mathrm{Gy}}^{-1}$ and β=0.013±0.002 ${\mathrm{Gy}}^{-2}$ (α/β=31.1 Gy). The corresponding biological effect at 2 Gy, $E_{2}=-\ln S\left(2\right)$, was 0.659 (SRR), 0.754 (USC), and 0.850 (LQ).

The fitted global uncertainty scales were $u_{\text{SF}}=0.175$ (SRR), 0.164 (USC), and 0.138 (LQ). The corresponding AIC values were −0.49 (SRR), 0.21 (USC), and −5.17 (LQ); the LQ‐model achieved the lowest AIC.

### 3.2 TCP curves from clinical data

**FIGURE 2:**
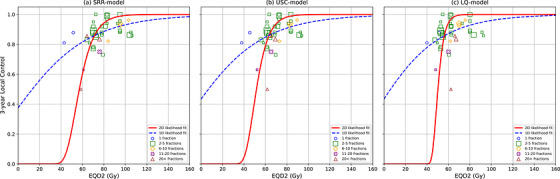
Comparison of model fits to clinical LC data. (a) SRR‐model, (b) USC‐model, and (c) LQ‐model fitted to 3‐year LC rates across different fractionation schemes using the 2D maximum‐likelihood framework (red solid curves). Blue dashed curves represent the conventional 1D likelihood fit for reference (Appendix C). Marker size is proportional to patient numbers. Data points are categorized by fraction number: 1 (blue circles), 2–5 (green squares), 6–10 (orange diamonds), 11–20 (purple crosses), and >20 (brown triangles).

All three models captured the sigmoidal dose–response relationship (Figure 2). Parameter estimates and performance metrics are summarized in Table 1. Across the three models, achieving 95% LC required approximately 1.4 (SRR), 1.4 (USC), and 1.2 (LQ) times the EQD2 of 50% LC.

**TABLE 1:** Summary of clinical TCP fits.

Parameter	SRR‐model	USC‐model	LQ‐model
$E_{2}$	0.659	0.754	0.850
$N_{0}$	$1.04\times 10^{8}$	$1.96\times 10^{8}$	$1.28\times 10^{9}$
m	3.08	2.92	1.68
${\mathrm{TCD}}_{50}$ (Gy)	57.1	51.6	50.2
${\mathrm{TCD}}_{95}$ (Gy)	81.4	71.8	60.5
$u_{\text{EQD2}}$	0.166	0.169	0.105
d (cm)	2.6 [2.0, 3.3]	3.2 [2.4, 4.1]	6.0 [4.6, 7.6]
${\mathrm{AIC}}_{\text{clinical}}$	142.9	133.4	117.6

$E_{2}=-\ln S\left(2\right)$: biological effect at 2 Gy; $N_{0}$: fitted clonogenic cell number; m: slope correction factor; TCD: tumor control dose (in EQD2); $u_{\text{EQD2}}$: effective dose‐axis uncertainty; d: predicted tumor diameter mean [P10, P90] from $N_{0}$; ${\mathrm{AIC}}_{\text{clinical}}$: 2D Mahalanobis orthogonal‐projection AIC.

The intrinsic scatter parameter was estimated as $\sigma_{\mathrm{int}}=0.020$ for SRR and USC, and 0.048 for LQ. Of note, for SRR and USC, the AIC profile was essentially flat (ΔAIC ≤2) over $\sigma_{\mathrm{int}}\in \left[0,0.02\right]$. However, the fitted curve degenerated toward a near‐step function as $\sigma_{\mathrm{int}}\to 0$. We therefore report the solution at $\sigma_{\mathrm{int}}=0.02$, the upper edge of this plateau. The resulting $\sigma_{\mathrm{LC}}$ values across cohorts span [0.006,0.082] for SRR, [0.006,0.082] for USC, and [0.009,0.082] for LQ. The mean relative LC‐axis uncertainty $\left\langle \sigma_{\mathrm{LC}}/\mathrm{LC}\right\rangle$ and the relative dose‐axis uncertainty $u_{\mathrm{EQD}2}$ were respectively 5.0% and 16.6% for SRR, 5.0% and 16.9% for USC, and 5.8% and 10.5% for LQ.

The fitted $N_{0}$ values progressively increased across the three models (Table 1), spanning more than one order of magnitude. This monotonic increase mirrors the ordering of $E_{2}$ values, reflecting the compensatory relationship between $E_{2}$ and $N_{0}$ in the TCP fit. The predicted tumor diameters provide an independent check on these $N_{0}$ estimates: the SRR and USC models both lie within approximately one standard deviation of the clinical reference 2.7±0.6 cm, while the LQ‐model exceeds the reference by more than five standard deviations.

### 3.3 Decomposition of TCP variability

Table 2 summarizes the backward‐inference decomposition of the total TCP exponent variability $\sigma_{\phi }^{2}$ into two components: clonogenic cell variability ($\sigma_{\ln N}$), estimated independently from external tumor volume data; and inter‐patient radiosensitivity variability ($\sigma_{E_{2}}$), derived via Equation (35).

**TABLE 2:** Decomposition of TCP exponent variability.

Model	$\sigma_{\phi }$	$\sigma_{\ln N}$	$\left({\mathrm{TCD}}_{50}/2\right)\sigma_{E_{2}}$	$\sigma_{E_{2}}$	${\mathrm{CV}}_{{\mathrm{SF}}_{2}}^{\mathrm{eff}}$
SRR‐model	3.35	0.60	3.30	0.116	0.116
USC‐model	3.16	0.60	3.10	0.120	0.120
LQ‐model	1.56	0.60	1.44	0.057	0.057

$\sigma_{\phi }$: TCP exponent variability; $\sigma_{\ln N}$: clonogenic cell variability; $\sigma_{E_{2}}$: inter‐patient radiosensitivity variability; ${\mathrm{CV}}_{{\mathrm{SF}}_{2}}^{\mathrm{eff}}$: effective coefficient of variation of ${\mathrm{SF}}_{2}$.

The clonogenic cell variability was estimated as $\sigma_{\ln N}=0.60$ from the between‐study standard deviation of lnV using 21 representative tumor volumes reported in Table S‐1, contributing approximately 3% of $\sigma_{\phi }^{2}$ for SRR and USC, and 15% for LQ. The remaining variance was attributed to inter‐patient radiosensitivity heterogeneity, yielding backward‐inferred $\sigma_{E_{2}}=0.116$ (SRR), 0.120 (USC), and 0.057 (LQ), all within the CLARIFi inter‐cell‐line bound of 0.255.

## 4 DISCUSSION

### 4.1 Cell survival model performance

The estimated $u_{\text{SF}}$ values were 0.175 (SRR), 0.164 (USC), and 0.138 (LQ). These estimates lie moderately below the inter‐assay CV of ${\mathrm{SF}}_{2}$ for A549 (approximately 21% across 192 publications),^36^ supporting the plausibility of the co‐estimated uncertainty scale. This global‐scale formulation was adopted in part because the per‐point uncertainties reported in the dataset do not follow the pattern expected for independent clonogenic replicates (smallest SDs at 10 and 12 Gy where variability should be highest), motivating a fit that does not rely on those individual SDs.

The USC‐model parameters (α=0.316 ${\mathrm{Gy}}^{-1}$, β=0.030 ${\mathrm{Gy}}^{-2}$, α/β=10.4 Gy) were consistent with the clinically accepted range for NSCLC and with those reported by Saga et al.^23^ (α/β=10.71±2.85 Gy). The transition dose $d_{t}=5.146$ Gy (95% profile likelihood CI: [4.80, 5.53] Gy) was lower than their reported value (6.160 Gy). This discrepancy is attributable to their use of an informative prior for $d_{t}$ derived from external cell lines, which propagated into their α and β estimates during simultaneous Bayesian inference. In contrast, our hierarchical fitting strategy—anchoring α and β from the low‐dose region before estimating $d_{t}$—does not invoke an informative prior. The need for such constraints reflects the inherent coupling between $d_{t}$ and α/β: the LQ‐model, which lacks $d_{t}$ entirely, achieved the lowest AIC (−5.17), with SRR‐model (−0.49) and USC‐model (0.21) yielding statistically comparable fits (ΔAIC ≤2). This suggests that the additional $d_{t}$ parameter is not statistically supported by the present data (4≤ΔAIC).

However, this apparent statistical redundancy of $d_{t}$ does not imply that it is biologically unnecessary. Because the two‐parameter LQ‐model lacks a mechanism to decouple low‐dose curvature from high‐dose behavior, it flattens the curvature globally to fit high‐dose data. Consequently, the LQ‐model yields $E_{2}=0.850$, substantially overestimating cell killing at 2 Gy compared to the USC‐model (0.754) and SRR‐model (0.659). This $E_{2}$ overestimation by the LQ‐model propagates into the downstream TCP analysis, inflating the fitted clonogenic cell number $N_{0}$.

The SRR and USC models differ from the LQ formulation through distinct parameterizations: the SRR‐model incorporates saturable repair through the parameter R, while the USC‐model introduces a transition dose $d_{t}$ separating low‐dose and high‐dose regimes. The SRR‐model converged to R=0.053 (95% profile likelihood CI: [0, 0.330]); the saturable repair contribution in A549 is thus moderately but not tightly constrained by the present data.

### 4.2 Methodological comparison and validation

The 2D‐fitted TCP curves aligned with the central tendency of the clinical data. The fitted ${\mathrm{TCD}}_{50}$ and ${\mathrm{TCD}}_{95}$ values were 57.1 and 81.4 Gy (SRR), 51.6 and 71.8 Gy (USC), and 50.2 and 60.5 Gy (LQ), giving a ${\mathrm{TCD}}_{95}$/${\mathrm{TCD}}_{50}$ ratio of approximately 1.4, 1.4, and 1.2 respectively. The estimated relative uncertainties confirmed the dominance of dose‐axis uncertainty: $u_{\mathrm{EQD}2}$ (16.6%, 16.9%, and 10.5%) exceeded the mean relative LC‐axis uncertainty $\left\langle \sigma_{\mathrm{LC}}/\mathrm{LC}\right\rangle$ (5.0%, 5.0%, and 5.8%) by factors of 3.3, 3.4, and 1.8 respectively, supporting the premise that a 2D framework accounting for dose uncertainty is essential for TCP modeling.

Prior TCP modeling for early‐stage NSCLC has used a range of statistical approaches, primarily differing in their treatment of LC‐axis uncertainty. Lee et al.^4^ fitted extensive clinical data using a binomial‐$\chi^{2}$ approach—weighting each cohort's LC‐axis residual by binomial sampling variance only—and reported the USC‐model (${\mathrm{TCD}}_{50}\approx 51$ Gy and ${\mathrm{TCD}}_{95}\approx 67$ Gy in BED). The reported $\chi^{2}/\mathrm{ndf}$ of 7.2 indicates LC‐axis scatter substantially exceeding binomial sampling alone, highlighting a limitation of the binomial‐$\chi^{2}$ approach. Guckenberger et al.^1^ adopted a statistically more rigorous LC‐axis treatment with Bayesian logistic regression and reported extremely gradual TCP for the LQ‐model, with ${\mathrm{TCD}}_{50}$ near 0 Gy and ${\mathrm{TCD}}_{95}\approx 236$ Gy (BED), a clinically implausible ${\mathrm{TCD}}_{50}$ that Santiago et al.^2^ attributed to identification difficulty of the curve initiation position in SBRT‐biased samples. Addressing this concern, Santiago et al.^2^ fitted a logistic model by nonlinear least squares with likelihood profiling for confidence intervals and the likelihood ratio test for model comparison, combining conventional fractionation data and reporting USC‐model ${\mathrm{TCD}}_{50}\approx 45$ Gy and ${\mathrm{TCD}}_{95}\approx 184$ Gy (BED). These likelihood‐based approaches improve LC‐axis treatment over binomial‐$\chi^{2}$ weighting, yet the dose axis remains treated as error‐free in all of them; consequently, dose‐axis variability appears as intrinsic curve flatness, yielding systematically shallow TCP curves. The present 2D framework addresses this remaining limitation by additionally modeling dose‐axis uncertainty within the likelihood.

Indeed, 1D estimation in our framework (Figure 2, blue dashed curves) reproduces this flattening pattern: it yielded ${\mathrm{TCD}}_{50}$ values of 13.4 Gy (SRR), 7.1 Gy (USC), and 6.3 Gy (LQ)—all clinically implausible for early‐stage NSCLC—with correspondingly inflated ${\mathrm{TCD}}_{95}$ values of 112.8, 103.1, and 92.7 Gy. These trends were consistent across random data splits (Appendix D).

We also compared our results against external large‐scale trials not included in our clinical data (Appendix E). Radiation Therapy Oncology Group (RTOG) 7301,^37^ a trial in locally advanced NSCLC, reported crude in‐field LC rates of 42%, 49%, and 65% at 40, 50, and 60 Gy. For all three models, the observed LC fell near the 2D‐fit TCP curves, whereas the 1D‐fit curves systematically overestimated LC, consistent with the flattening tendency of 1D estimation discussed above. In contrast, the SBRT trials (RTOG 0236, RTOG 0618, RTOG 0813)^38^, ^39^, ^40^ all reported 3‐year LC exceeding 85%, falling in the plateau region where the 2D and 1D predictions are both high and the practical distinction between the two approaches diminishes. Geometrically, within the 2D framework, each clinical cohort contributes an elliptical uncertainty cloud around the fitted TCP curve, with widths of approximately 5.0%–5.8% along the LC axis and 10.5%–16.9% along the dose axis, and the fit is identified as the curve passing through the joint centers of these clouds. Nevertheless, the 2D‐fitted curves appear somewhat steeper than the external trial data would suggest. This likely reflects the absence of clinical cohorts in the transition region (LC < 0.5, below ${\mathrm{TCD}}_{50}$), leaving curve steepness primarily determined by the high‐EQD2 plateau; we acknowledge this as a limitation.

In addition to the external clinical‐outcome validation above, the predicted tumor diameter distributions provide an independent check on the fitted $N_{0}$ values. Modeling inter‐patient $N_{0}$ heterogeneity as log‐normal—motivated by $N_{0}=\rho V$ with constant clonogen density ρ—yields mean diameters of 2.6±0.5 cm (SRR), 3.2±0.6 cm (USC), and 6.0±1.2 cm (LQ), compared to 2.7±0.6 cm from the same external tumor volume data. The SRR and USC models both fall within approximately one standard deviation of the clinical reference, supporting the framework's translational validity. The LQ‐model substantially overestimates the diameter, consistent with its inflated $E_{2}$. Alternative approaches using exponential^41^, ^42^ or normal^43^ distributions for $N_{0}$ heterogeneity have been proposed; comparing their impact on TCP predictions using the same clinical datasets is left to future work. Note also that $\sigma_{\ln N}=0.60$ represents a lower bound on the true inter‐patient variability, as it was estimated from between‐study variances of aggregated tumor volumes under a constant‐ρ assumption. The clinical reference of 2.7 cm is a cross‐study average of per‐study mean tumor volumes and is therefore unaffected by ρ variability. By contrast, the predicted mean diameter increases with $\sigma_{\ln N}$ for a log‐normal $N_{0}$ distribution.

### 4.3 Mechanistic decomposition of TCP steepness

An important observation from the variance decomposition is that despite the large absolute clonogenic burden and its substantial inter‐patient variability (coefficient of variation ∼66%), the contribution of clonogenic heterogeneity to TCP steepness is small: the externally constrained $\sigma_{\ln N}=0.60$ contributes only 3%–15% to the total variance $\sigma_{\phi }^{2}$ across all three models. Under this minimal decomposition, the dose–response variability $\left({\mathrm{TCD}}_{50}/2\right)\sigma_{E_{2}}$ accounts for the remaining 85%–97% of $\sigma_{\phi }^{2}$ across the three models (Table 2). These findings align with and extend prior work by Roberts and Hendry,^43^ Buffa et al.,^44^ and Stavreva et al.,^45^ who highlighted inter‐patient radiosensitivity heterogeneity as a major driver of TCP curve steepness in LQ‐based models. Our analysis confirms this conclusion for the LQ‐model and extends it to the SRR and USC models, which incorporate dedicated high‐dose parameterizations.

The backward‐inferred inter‐patient radiosensitivity variability yielded $\sigma_{E_{2}}$ = 0.057–0.120 across the three models, all below the CLARIFi‐derived inter‐cell‐line $\sigma_{E_{2}}$ bound of 0.255 (across 41 NSCLC cell lines^24^). One possible interpretation is that the NSCLC tumors comprising these early‐stage clinical cohorts exhibit a narrower inter‐patient ${\mathrm{SF}}_{2}$ variability than the genetically distinct cell lines spanning the CLARIFi panel. An alternative possibility, related to the data coverage limitation noted in Section 4.2, is that the TCP slope may be overestimated due to the absence of clinical observations with LC < 0.5. Under this scenario, the inferred $\sigma_{E_{2}}$ would correspondingly be underestimated. The observed AIC plateau in $\sigma_{\mathrm{int}}\in \left[0,0.02\right]$ for SRR and USC likely reflects this same data limitation: in the absence of observations in the steepness‐defining region, $\sigma_{\mathrm{int}}$ and $u_{\mathrm{EQD}2}$ trade off freely within the plateau without unique identification.

Among the three models, the LQ‐model produced the smallest $\sigma_{\phi }$ and $\sigma_{E_{2}}$. This pattern reflects how the inflated α/β=31.1 Gy of the LQ‐model affects EQD2. For multi‐fraction (≥6 fr) regimens, this α/β compresses the EQD2 range to 48–76 Gy, substantially tighter than under SRR or USC. This narrow EQD2 range coincides with the dense region of SBRT cohorts (50–65 Gy). The compression arises from Equation (19): the dose‐modifying ratio approaches unity as α/β grows, limiting EQD2 amplification at high dose‐per‐fraction. This tight clustering allows the steep TCP fit. Although the LQ‐model achieves the lowest AIC in both cell‐survival fitting (4≤ΔAIC) and clinical‐data fitting (10<ΔAIC), it exhibits structural fragility at both extremes: in the 1D likelihood framework, the curve is overly flat, collapsing to a degenerate solution; in the 2D likelihood framework, the curve is overly steep, approaching a near‐step‐function shape. The LQ‐model thus sits between two degenerate regimes, suggesting its statistical advantage masks an underlying structural limitation.

A related perspective comes from Díaz Hernández et al.,^41^ who sought to explain TCP curve steepness in NSCLC by introducing inter‐patient heterogeneity in the radiosensitivity parameter α of the LQ‐model (mathematically equivalent to $E_{2}$ in the LQ context up to a constant offset). Among the distributions they tested, a uniform distribution provided the best fit to clinical data, in preference to a Gaussian distribution. The CLARIFi database,^24^ however, shows ${\mathrm{mSF}}_{2}$ to be approximately Gaussian; via the small‐variance linearization $E_{2}=-\ln{\mathrm{SF}}_{2}$, the implied $\sigma_{E_{2}}$ distribution is likewise approximately Gaussian, consistent with our backward‐inference assumption. Díaz Hernández et al. themselves noted that the choice of distribution shape is an ad hoc premise; their analysis treated dose, volume, treatment time, and follow‐up time as known inputs and minimized a 1D likelihood of observed versus predicted LC. Our findings shed new light on this premise: rather than indicating that in vitro Gaussian ${\mathrm{SF}}_{2}$ distributions become genuinely uniform in vivo, the uniform‐distribution preference may partly reflect compensation for the curve‐flattening EIV bias inherent to 1D analysis, where a broader (uniform) α distribution introduces additional flattening.

As described in Section 2.6, we adopted $\mathrm{Cov}(\ln N,E_{2})=0$ and $\sigma_{\mathrm{meta}}^{2}=0$ as minimal‐model assumptions. Nonzero values would shift the inferred $\sigma_{E_{2}}$: a positive $\mathrm{Cov}(\ln N,E_{2})$ would increase it, whereas a negative Cov or a positive $\sigma_{\mathrm{meta}}^{2}$ would reduce it. A plausible mechanism for negative Cov is volume‐dependent hypoxia: larger gross tumor volumes tend to harbor larger hypoxic subvolumes,^46^, ^47^ which exhibit reduced effective radiosensitivity.^48^ A nonzero $\sigma_{\mathrm{meta}}^{2}$ would absorb biological effects beyond classical clonogenic cell kill, such as vascular damage^49^ and immune‐mediated effects^50^ that are particularly relevant at high doses per fraction. The fact that all three models yield $\sigma_{E_{2}}$ within the CLARIFi bound under the minimal assumptions supports the framework's tenability without invoking these additional contributions; explicit estimation of Cov and $\sigma_{\mathrm{meta}}^{2}$ would require data beyond the present analysis.

### 4.4 Limitations

A practical caveat of the translational framework is its dependence on a single NSCLC cell line. A549 was selected because its ${\mathrm{mSF}}_{2}$ in the CLARIFi database^24^ closely matches the NSCLC‐wide median, providing a representative central tendency. The same cell line, however, exhibits a systematic offset between the Saga et al.^23^ measurement (${\mathrm{SF}}_{2}=0.49$) and the CLARIFi value (${\mathrm{SF}}_{2}=0.58$), corresponding to $\Delta E_{2}=-23.5\%$. To assess the propagation of this specific offset into the TCP fit, we performed an empirically anchored sensitivity analysis (Appendix F). For SRR, the clinically relevant dose targets ${\mathrm{TCD}}_{50}$ and ${\mathrm{TCD}}_{95}$ shifted by up to ∼9%; for USC and LQ, where the EQD2 conversion was preserved by construction, the shifts were within ∼1%, confirming the robustness of the clinical dose targets. Correspondingly, $\sigma_{\phi }$ at ${\mathrm{TCD}}_{50}$ decreased to compensate for the reduced $E_{2}$, with the backward‐inferred $\sigma_{E_{2}}$ correspondingly decreased; the fitted TCP curves were therefore essentially shape‐invariant in the clinically relevant range. The fitted $N_{0}$, in contrast, decreased by approximately 2 orders of magnitude, indicating that the absolute clonogenic burden estimate is sensitive to the assumed cell‐killing efficiency at 2 Gy.

A more fundamental limitation lies in the upstream experimental data itself. A good model fit does not exclude the possibility of systematic biases, and several critical experimental details of the Saga et al.^23^ dataset are unreported, including seeding density, dissociation protocols, and a relatively short pre‐irradiation adhesion time. Additionally, 60 mm dishes were used uniformly across all dose levels, whereas larger vessels are conventionally employed at high doses to maintain low initial confluence. At high doses (≥10 Gy), the net bias direction is a priori unclear: incomplete dissociation may inflate apparent survival via biological multiplicity, while bystander and confluence‐related effects may act in the opposite direction. These potential biases cannot be quantified from the published data alone, and their propagation into the downstream TCP analysis represents an inherent limitation of any translational study relying on external cell‐survival measurements.

Finally, the repopulation correction used to convert prescriptions to EQD2 relies on fixed literature‐based values for the kick‐off time $T_{k}$ and the tumor doubling time $T_{TD}$, and the sensitivity of the TCP fits to these assumed repopulation parameters was not explored.

## 5 CONCLUSIONS

We developed a 2D maximum‐likelihood framework for TCP modeling that explicitly integrates uncertainties in both dose and LC. By mitigating the EIV bias inherent to conventional 1D fitting, this approach avoided artefactual curve flattening and generated clinically plausible predictions aligned with the central tendency of multi‐institutional data for early‐stage NSCLC. The dose‐axis uncertainty consistently exceeded the LC‐axis uncertainty across all three models, justifying its explicit treatment in TCP analyses.

Beyond reducing bias, the backward‐inference decomposition revealed that TCP curve steepness is dominated by inter‐patient radiosensitivity variability, with clonogenic cell‐number variability contributing negligibly. The inferred radiosensitivity variability lay within the CLARIFi inter‐cell‐line bound, demonstrating that clinical TCP steepness is consistent with in vitro radiosensitivity heterogeneity and advancing TCP modeling from phenomenological curve fitting toward a mechanistic interpretation.

## FUNDING INFORMATION

This research did not receive any specific grant from funding agencies in the public, commercial, or not‐for‐profit sectors.

## CONFLICT OF INTEREST STATEMENT

The authors declare no conflicts of interest related to this study.

## Supporting information

Supporting Information

## Figures and Tables

**FIGURE B1:**
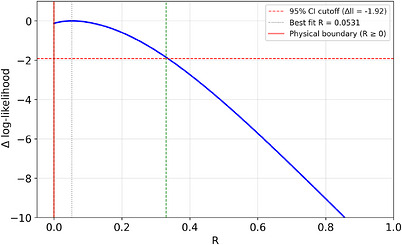
Profile likelihood for the SRR‐model repair parameter R. Dashed horizontal line: 95% CI cutoff. Best‐fit R=0.053; 95% CI: [0.000, 0.330].

**FIGURE B2:**
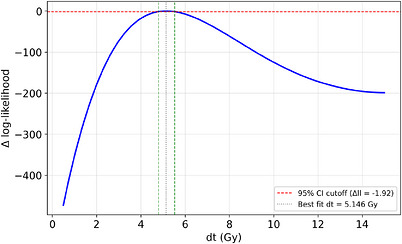
Profile likelihood for the USC‐model transition dose $d_{t}$. Dashed horizontal line: 95% CI cutoff. Best‐fit $d_{t}=5.146$ Gy; 95% CI: [4.80, 5.53] Gy.

**FIGURE E1:**
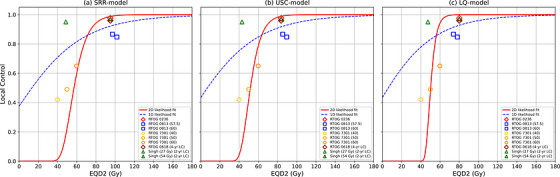
External consistency check of 2D (red solid) and 1D (blue dashed) TCP curves against prospective trial outcomes. (a) SRR‐model, (b) USC‐model, and (c) LQ‐model. Annotations in parentheses indicate non‐3‐year endpoints.

## Data Availability

This study used only previously published data. The clinical data extracted from the literature are provided in the Supplementary Materials. The cell survival data were obtained from Saga et al.,^23^ and the ${\mathrm{SF}}_{2}$ variability data were obtained from the CLARIFi database.^24^

## APPENDIX A 2D MAXIMUM‐LIKELIHOOD FRAMEWORK

This appendix provides the full derivation of the 2D maximum‐likelihood formula used for TCP curve fitting (Section 2.4.2).

### A.1 Bivariate gaussian likelihood and AIC

For each cohort i at observation $\left(\mathrm{EQD}2_{o,i},\;{\mathrm{LC}}_{o,i}\right)$, the reference point on the TCP curve is obtained by orthogonal projection in the 2D Mahalanobis metric:

$$\begin{array}{cc} x_{i}^{*}\left(u_{\mathrm{EQD}2}\right) & =\arg\min_{x}\;\frac{{\left(\mathrm{EQD}2_{o,i}-x\right)}^{2}}{\sigma_{x,i}^{2}}\\ & \;+\frac{{\left({\mathrm{LC}}_{o,i}-\mathrm{TCP}\left(x;\hat{\theta }\right)\right)}^{2}}{\sigma_{y,i}^{2}},\;y_{i}^{*}=\mathrm{TCP}\left(x_{i}^{*};\hat{\theta }\right). \end{array}$$

The dose‐axis standard deviation is taken proportional to cohort EQD2, $\sigma_{x,i}=u_{\mathrm{EQD}2}\;\mathrm{EQD}2_{o,i}$, and the LC‐axis variance is given by the joint‐floor formula (Equation 24):

$$\sigma_{y,i}^{2}=\sigma_{\mathrm{Jeff},i}^{2}+\max\;\left(0,\;\sigma_{\mathrm{int}}^{2}-{\left({\mathrm{TCP}}^{\prime }\left(\mathrm{EQD}2_{o,i}\right)\;\sigma_{x,i}\right)}^{2}\right).$$

Under independent Gaussian errors, the bivariate likelihood is

$$\begin{array}{cc} & L\left(u_{\mathrm{EQD}2}\right)=\prod_{i=1}^{n}\frac{1}{2\pi \;\sigma_{x,i}\sigma_{y,i}}\\ & \;\times \exp\;\left(-\frac{{\left(\mathrm{EQD}2_{o,i}-x_{i}^{*}\right)}^{2}}{2\sigma_{x,i}^{2}}-\frac{{\left({\mathrm{LC}}_{o,i}-y_{i}^{*}\right)}^{2}}{2\sigma_{y,i}^{2}}\right). \end{array}$$

The log‐likelihood expands to

$$\ln L=-n\ln\left(2\pi \right)-\sum_{i=1}^{n}\ln\sigma_{x,i}-\sum_{i=1}^{n}\ln\sigma_{y,i}-\frac{1}{2}\sum_{i=1}^{n}d_{\mathrm{orth},i}^{2},$$

where $d_{\mathrm{orth},i}^{2}$ is the squared 2D Mahalanobis distance from the observation to its orthogonal projection. The AIC follows as

$$\begin{array}{cc} \mathrm{AIC} & =2k-2\ln\hat{L}=2k+2n\ln\left(2\pi \right)+2\sum_{i=1}^{n}\ln\sigma_{x,i}\\ & \;+2\sum_{i=1}^{n}\ln\sigma_{y,i}+\sum_{i=1}^{n}d_{\mathrm{orth},i}^{2}, \end{array}$$

with k=4 (all parameters $\theta =(N,\;m,\;u_{\mathrm{EQD}2},\;\sigma_{\mathrm{int}})$ jointly estimated). The fitted $u_{\mathrm{EQD}2}$ reflects cohort scatter perpendicular to the fitted curve. The use of joint‐floor $\sigma_{y}$ ensures that the intrinsic *Y*‐side scatter $\sigma_{\mathrm{int}}$ is not absorbed into $u_{\mathrm{EQD}2}$; only the residual scatter unexplained by $\sigma_{\mathrm{Jeff}}$ and $\sigma_{\mathrm{int}}$ contributes to the dose‐axis allocation.

(correlated errors) If correlation between axes is to be modeled, replace the independent form by

$$L=\prod_{i=1}^{n}\frac{1}{2\pi \sqrt{|\Sigma_{i}|}}\exp\;\left(-\frac{1}{2}r_{i}^{⊤}\Sigma_{i}^{-1}r_{i}\right),\;r_{i}=\left[\begin{array}{c} \mathrm{EQD}2_{o,i}-x_{i}^{*}\\ {\mathrm{LC}}_{o,i}-y_{i}^{*} \end{array}\right],$$

with $\Sigma_{i}$ the 2×2 covariance matrix; the AIC then replaces $\ln\sigma_{x,i}+\ln\sigma_{y,i}$ by $\frac{1}{2}\ln\left|\Sigma_{i}\right|$ and the sum of squared terms by the Mahalanobis quadratic form. This extension is not used in the present analysis.

## APPENDIX B PROFILE LIKELIHOOD CONFIDENCE INTERVALS FOR R and $d_{t}$

For the repair parameter R of the SRR‐model and the transition dose $d_{t}$ of the USC‐model, the likelihood surfaces are substantially asymmetric, and we therefore report profile likelihood confidence intervals.

The profile likelihood was computed by fixing the parameter of interest at a grid of values and re‐optimizing the remaining parameters at each grid point. The 95% confidence interval is defined by the region satisfying $2[\ln{\hat{L}}_{\max}-\ln{\hat{L}}_{\text{profile}}\left(\theta_{j}\right)]<3.841$. For $d_{t}$, consistent with the hierarchical fitting strategy (Section 2.4.1), the profile likelihood is evaluated with α and β held at their low‐dose estimates; only $u_{\text{SF}}$ is re‐optimized at each grid value of $d_{t}$.

Figure B1 shows the profile likelihood for R. The lower bound of the CI reaches the physical boundary (R=0), confirming that the data are consistent with nearzero repair capacity. Figure B2 shows the profile likelihood for $d_{t}$, yielding a well‐defined CI of [4.80, 5.53] Gy under the hierarchical approach with α and β fixed.

## APPENDIX C 1D LIKELIHOOD AND FIT PARAMETERS

For comparison with the 2D formulation, we also fitted a 1D likelihood in which the dose axis is treated as deterministic and the entire LC‐axis residual scatter is absorbed into a single fitted scale. The dose‐axis uncertainty is omitted ($\sigma_{x}=0$) and the *LC*‐axis variance is given by

(C1)
$$\sigma_{y,i}^{2,\;(1D)}=\sigma_{\mathrm{Jeff},i}^{2}+{\left(u_{\mathrm{LC}}\;{\mathrm{LC}}_{o,i}\right)}^{2},$$

with free parameters $\theta^{\left(1D\right)}=\left(N,\;m,\;u_{\mathrm{LC}}\right)$ (k=3), where $u_{\mathrm{LC}}\in \left[0.01,\;1.00\right]$. Optimization followed the same protocol as in Section 2.4.2.

Table C1 summarizes the resulting parameters. The 1D fit yields ${\mathrm{TCD}}_{50}$ values of 6–13 Gy and clonogenic burdens $N_{0}$ of order $10^{1}$; for USC and LQ, $N_{0}$ converged to the lower optimization bound.

**TABLE C1:** TCP parameters of the 1D likelihood framework.

Parameter	SRR‐model	USC‐model	LQ‐model
$N_{0}$	$5.80\times 10^{1}$	$1.01\times 10^{1}$	$1.00\times 10^{1}$
m	12.61	13.93	14.13
${\mathrm{TCD}}_{50}$ (Gy)	13.4	7.1	6.3
${\mathrm{TCD}}_{95}$ (Gy)	112.8	103.1	92.7
$u_{\mathrm{LC}}$	0.066	0.068	0.073

$N_{0}$: fitted clonogenic cell number; m: slope correction factor; TCD: tumor control dose (in EQD2); $u_{\mathrm{LC}}$: LC‐axis uncertainty scale.

## APPENDIX D ROBUSTNESS TO CLINICAL DATASET COMPOSITION

To examine whether the flattening of the 1D likelihood framework relative to the 2D framework depends on the composition of the clinical dataset, we repeated the random‐split analysis over 20 independent seeds (10 replicate splits each; in every split, 30 of the 41 clinical data points were randomly selected for fitting and the remaining 11 excluded), giving 200 fits per model and framework. Regardless of the data subset, the 2D likelihood framework preserved the sigmoid‐shaped TCP dose–response, whereas the 1D likelihood framework reproduced the same flattened response. Although the individual split estimates varied (Table D1), reflecting the small 30‐point subsets, this qualitative distinction was consistent across all 20 seeds.

**TABLE D1:** 2D and 1D ${\mathrm{TCD}}_{50}$/${\mathrm{TCD}}_{95}$ estimates across 20 random‐split seeds.

		**2D likelihood**		**1D likelihood**	
Model	Metric	Split mean ± SD	Full dataset	Split mean ± SD	Full dataset
SRR	${\mathrm{TCD}}_{50}$ (Gy)	50.5±15.3	57.1	17.4±11.7	13.4
	${\mathrm{TCD}}_{95}$ (Gy)	84.4±8.5	81.4	109.7±7.7	112.8
USC	${\mathrm{TCD}}_{50}$ (Gy)	46.7±13.5	51.6	10.7±7.9	7.1
	${\mathrm{TCD}}_{95}$ (Gy)	73.8±7.0	71.8	100.6±6.3	103.1
LQ	${\mathrm{TCD}}_{50}$ (Gy)	49.3±5.4	50.2	6.6±1.6	6.3
	${\mathrm{TCD}}_{95}$ (Gy)	60.8±2.8	60.5	92.6±4.9	92.7

Split mean ± SD over 20 seeds × 10 splits (200 fits per cell), each split fitting 30 of the 41 points (11 excluded). Full‐dataset estimates from Tables 1 and C1 are shown for reference.

## APPENDIX E EXTERNAL CONSISTENCY CHECK

To assess the clinical plausibility of the 2D and 1D TCP estimates, we compared model predictions against prospective trial outcomes not included in our clinical dataset. The representative dose per fraction was taken as the PTV peripheral dose. For the RTOG SBRT protocols (0236,^38^ 0618,^39^ and 0813^40^), the prescription dose serves as the PTV peripheral dose by protocol design. For RTOG 7301^37^ (conventional fractionation), the dose per fraction of 2 Gy was used directly. Table E1 summarizes the trial data and model‐predicted TCP values, and Figure E1 shows the data points overlaid on the 2D and 1D TCP curves. The two frameworks should be interpreted differently. Under the 1D framework, the deviation of a data point from the curve is measured only along the *LC*‐axis. Under the 2D framework, the deviation is measured jointly along both axes.

**TABLE E1:** LC and EQD2 for the external prospective trials.

				SRR	USC	LQ
Trial	Regimen	LC	Type	EQD2	EQD2	EQD2
RTOG 7301^37^	40 Gy/20 fr	42%	∼3‐yr	39.92	39.93	39.94
RTOG 7301^37^	50 Gy/25 fr	49%	∼3‐yr	49.82	49.85	49.86
RTOG 7301^37^	60 Gy/30 fr	65%	∼3‐yr	59.73	59.76	59.79
RTOG 0236^38^	54 Gy/3 fr	97.6%	3‐yr	94.94	83.67	80.09
RTOG 0618^39^	54 Gy/3 fr	96%	4‐yr	94.94	83.67	80.09
RTOG 0813^40^	57.5 Gy/5 fr	86.7%	3‐yr	97.07	85.25	74.00
RTOG 0813^40^	60 Gy/5 fr	84.7%	3‐yr	101.78	89.42	78.12
Singh^51^	54 Gy/3 fr	97%	2‐yr	94.94	83.67	80.09
Singh^51^	27 Gy/1 fr	95%	2‐yr	48.59	42.90	47.38

EQD2 values in Gy. RTOG 0813 data are from central tumors. Prescription doses were used directly for the model‐based EQD2 calculation, with the repopulation correction applied.

## APPENDIX F SENSITIVITY TO A549 SYSTEMATIC OFFSET

While A549 is retained as the NSCLC representative on the basis of its ${\mathrm{mSF}}_{2}$ matching the NSCLC‐wide median, the same cell line exhibits a systematic offset between the Saga et al.^23^ measurement (${\mathrm{SF}}_{2}=0.49$, $E_{2}=0.72$) and the CLARIFi A549 ${\mathrm{mSF}}_{2}$ (0.58, $E_{2}=0.55$).^24^ This corresponds to a systematic difference of −15.7% in ${\mathrm{SF}}_{2}$, reflecting inter‐laboratory measurement variability of the same cell line. To assess the propagation of this offset into the TCP fit, we performed a sensitivity check at $\Delta E_{2}=-23.5\%$ for all three models. This is an empirically anchored sensitivity analysis tied to the specific Saga–CLARIFi systematic offset for A549; it is not a comprehensive sensitivity analysis spanning the full plausible range of $E_{2}$ uncertainty.

For each model, the cell‐survival parameters were rescaled to produce the target $E_{2}$ value while preserving the EQD2 conversion: for the SRR‐model, the linear killing parameter a was solved numerically with R fixed; for the USC and LQ models, α was rescaled with α/β held at 10.4 Gy and 31.1 Gy, respectively. The TCP curve was re‐fitted using the 2D maximum‐likelihood framework, and the variance decomposition was recomputed.

Table F1 summarizes the results. For SRR, the clinically relevant dose targets ${\mathrm{TCD}}_{50}$ and ${\mathrm{TCD}}_{95}$ shifted by up to ∼9%, reflecting a procedural constraint of the rescaling itself, which adjusted only the linear killing parameter a (with R fixed), so the EQD2 conversion could not be kept exactly invariant. For USC and LQ, where α/β was held fixed and the EQD2 conversion was preserved by construction, the shifts were within ∼1%, confirming the robustness of the clinical dose targets. The fitted $N_{0}$, however, decreased by approximately 1.7–2.2 orders of magnitude across the three models, reflecting its strong dependence on the assumed cell‐killing efficiency at 2 Gy and shifting the implied tumor diameter distributions toward sub‐clinical values (d≲1.2 cm). Correspondingly, $\sigma_{\phi }$ at ${\mathrm{TCD}}_{50}$ decreased to compensate for the reduced $E_{2}$, with the backward‐inferred $\sigma_{E_{2}}$ correspondingly decreased; the fitted TCP curves were therefore essentially shape‐invariant in the clinically relevant range.

**TABLE F1:** Sensitivity of TCP parameters to the A549 systematic offset.

Model	$\Delta E_{2}$ (%)	$E_{2}$	$N_{0}$	${\mathrm{TCD}}_{50}$ (Gy)	${\mathrm{TCD}}_{95}$ (Gy)	$\sigma_{\phi }$	$\sigma_{E_{2}}$	${\mathrm{CV}}_{{\mathrm{SF}}_{2}}^{\mathrm{eff}}$
SRR	—	0.659	$1.04\times 10^{8}$	57.1	81.4	3.35	0.116	0.116
	−23.5	0.504	$1.97\times 10^{6}$	58.9	88.7	3.11	0.104	0.104
USC	—	0.754	$1.96\times 10^{8}$	51.6	71.8	3.16	0.120	0.120
	−23.5	0.577	$1.99\times 10^{6}$	51.5	71.8	2.31	0.087	0.087
LQ	—	0.850	$1.28\times 10^{9}$	50.2	60.5	1.56	0.057	0.057
	−23.5	0.650	$8.73\times 10^{6}$	50.3	60.5	0.91	0.027	0.027

Cell‐survival parameters were rescaled to the target $E_{2}$ with the EQD2 conversion held fixed (SRR: a rescaled, R fixed; USC and LQ: α rescaled with α/β = 10.4 and 31.1 Gy, respectively).

The framework would be most strengthened by the availability of multiple A549 cell‐survival datasets spanning the low‐ to high‐dose range. Averaging across such datasets would yield $E_{2}$ and radiobiological model parameters more representative of the average NSCLC SF response, reducing the inter‐laboratory variability and thereby reinforcing the framework's translational validity.
