# Supplementary material for: Translational tumor control probability modeling for NSCLC: A two‐dimensional maximum‐likelihood framework
Source: Med Phys. 2026 Jul 31;53(8):e70619. doi: 10.1002/mp.70619 (PMC13425703; doi:10.1002/mp.70619)
Supplement: Supplementary file 1 — Supporting Information [file MP-53-0-s001.pdf]

Supplementary Material: Clinical Data Included in Analysis

Search Strategy

The systematic literature search was conducted in PubMed using the following search algorithm:

> ("lung neoplasms"[MH] OR "lung cancer"[TIAB] OR "non-small cell lung cancer"[TIAB] OR NSCLC[TIAB]) AND ("radiotherapy"[MH] OR radiation[TIAB] OR irradiation[TIAB] OR radiotherapy[TIAB] OR "radiation therapy"[TIAB] OR "Dose Fractionation, Radiation"[MH] OR hypofractionation[TIAB] OR hypofractionated[TIAB] OR "accelerated hypofractionation"[TIAB] OR hyperfractionated[TIAB]) AND ("local control"[TIAB] OR "tumor control"[TIAB] OR "local failure"[TIAB] OR "local relapse"[TIAB] OR "local progression"[TIAB] OR "local recurrence"[TIAB] OR "treatment outcome"[TIAB] OR "treatment outcomes"[TIAB] OR "treatment failure"[TIAB] OR survival[TIAB] OR "overall survival"[TIAB] OR mortality[TIAB] OR "quality of life"[TIAB] OR QOL[TIAB] OR QoL[TIAB]) AND ("stage I"[TIAB] OR "stage 1"[TIAB] OR "stage II"[TIAB] OR "stage 2"[TIAB] OR "early-stage"[TIAB] OR "early stage"[TIAB] OR "T1-3N0M0"[TIAB] OR "cT1-3N0M0"[TIAB]) NOT (Case Reports[pt] OR Review[pt] OR Meta-Analysis[pt] OR Systematic Review[pt] OR "technical report"[TIAB]) AND English[LA] AND hasabstract[text] AND ("2000/01/01"[DP] : "2021/12/31"[DP])

Study Selection Process

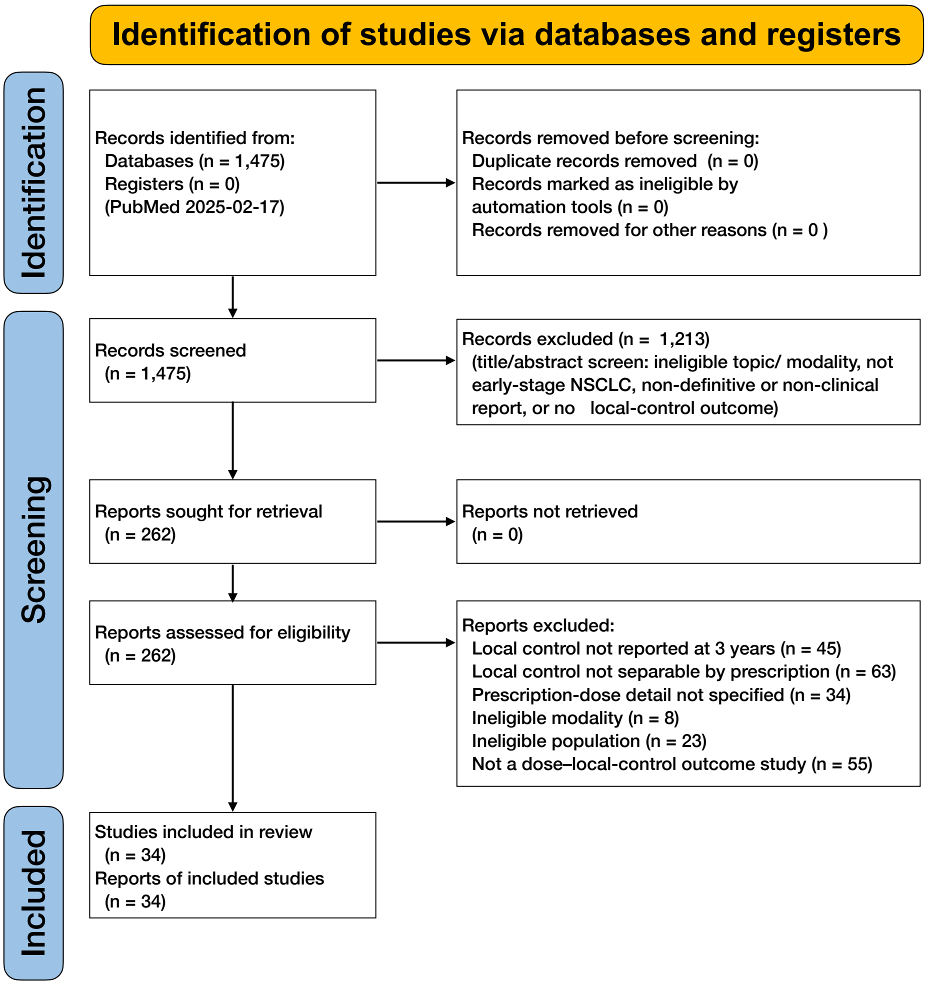

Figure S-1: PRISMA 2020 flow diagram showing the study selection process for the systematic literature search identifying early-stage NSCLC radiotherapy studies used as TCP model inputs

The search was executed on 17 February 2025 and returned 1,475 unique PubMed records. Records were screened by a single reviewer on title and abstract; 1,213 were excluded at this stage as off-topic by modality, disease stage, or treatment intent, or as lacking a local-control outcome. The remaining 262 reports were retrieved and assessed in full text against the inclusion criteria (early-stage NSCLC treated with definitive radiotherapy and reporting a dose-stratified ∼3-year local-control rate). Of these, 228 were excluded for the reasons enumerated in Figure S-1, leaving 34 studies (41 dose–local-control data points) for analysis. Data extraction was performed by a single reviewer; the extracted values were checked against the source reports on two occasions to minimize transcription errors. Because this single-reviewer process was not independently duplicated, the possibility that some eligible studies were missed cannot be excluded; the search was also restricted to a single database (PubMed), which may not capture studies indexed only in other sources. These are recognized limitations of the review process.

Risk-of-Bias Assessment

A formal risk-of-bias or study-quality appraisal (e.g., RoB 2, ROBINS-I, or QUADAS-2) was not performed. This work is a mechanistic radiobiological (tumor-control-probability) modeling analysis that uses published aggregate dose–local-control rates as model inputs; it is not a comparative-effectiveness or diagnostic-accuracy synthesis, and the aggregate control rates are not the type of comparative effect estimate that these instruments are designed to appraise. Study-level bias was instead mitigated by (i) confirming that the included studies represent distinct, non-overlapping patient cohorts, so that no cohort contributes more than once; and (ii) reporting the dataset-composition robustness analysis (Appendix D) of the main manuscript.

Clinical Data

Table S-1: Clinical data of early-stage NSCLC treated with radiotherapy.

Authors	N	Periph. Dose (Gy)	Frac.	LC-3y (%)	σ_{Jeff} (%)	d (cm)
Cheung	33	3.80	12	63	8.18	4.2
Langendijk	46	1.80	35	50	7.22	—
Nagata	32	10.50	4	95	4.19	—
Fritz	40	24.00	1	81	6.14	3.3
Baumann	57	15.00	3	92	3.67	3.1
Stephans	56	10.00	5	97	2.52	2.4
Brown	31	20.00	3	85.8	6.27	2.9
Fakiris	34	18.00	3	88	5.62	—
	36	19.80	3	88	5.46	—
Ricardi	62	15.00	3	87.8	4.18	2.4
Crabtree	76	18.00	3	89	3.61	2.0
Videtic	26	10.00	5	94	5.02	2.2
Haasbeek	63	7.50	8	92.6	3.38	3.6
Shibamoto	124	10.80	4	86	3.12	2.7
	52	11.70	4	73	6.07	2.7
Shirata	45	10.80	4	100	1.51	—
	29	6.75	8	82.1	7.04	—
Inoue	25	10.00	4	81	7.73	2.2
Rwigema	46	18.00	3	95.5	3.28	—
Shibamoto	180	10.80	4	85	2.66	—
Lindberg	57	15.00	3	92	3.67	—
Hayashi	81	10.50	4	91.8	3.10	—
Hamaji	104	10.50	4	77	4.10	2.5
Nagata	169	10.50	4	87.6	2.54	2.1
Navarro-Martin	38	13.50	4	94	4.07	—
Tsurugai	234	10.50	4	89	2.05	—
Shaverdian	110	18.00	3	100	0.63	—
Nyman	49	15.00	3	86.4	4.91	3.1
	53	1.90	35	85.7	4.81	3.1
Miyakawa	71	10.80	4	88	3.87	—
Swaminath	108	3.80	15	75	4.14	—
Lee	155	15.00	4	86.3	2.76	2.6
Cummings	98	8.00	5	83	3.79	—
Karasawa	56	10.50	4	78.2	5.47	2.5
	103	2.64	25	83.5	3.65	3.0
Menoux	90	7.50	8	94	2.57	—
Tsurugai	157	10.00	5	99	0.90	—
	66	10.00	5	100	1.04	—
Weiss	100	12.00	4	92	2.75	—
Alongi	29	6.65	10	96.2	4.04	2.0
	44	28.50	1	87.8	4.97	2.0

Note: Tumor diameter d represents mean or median values where reported; values converted from tumor volume assuming spherical geometry ($d = \sqrt[3]{6V/\pi}$). σ_{Jeff}: posterior standard deviation of the binomial proportion under a Jeffreys prior Beta(0.5, 0.5), computed from the reported LC rate and sample size of each study, expressed in the same percentage-point units as LC-3y.

References

[1] Cheung PC, Yeung LT, Basrur V, et al. Accelerated hypofractionation for early-stage non-small-cell lung cancer. *Int J Radiat Oncol Biol Phys.* 2002;54(4):1014-1023.

[2] Langendijk JA, Aaronson NK, de Jong JM, et al. Quality of life after curative radiotherapy in Stage I non-small-cell lung cancer. *Int J Radiat Oncol Biol Phys.* 2002;53(4):847-853.

[3] Nagata Y, Takayama K, Matsuo Y, et al. Clinical outcomes of a phase I/II study of 48 Gy of stereotactic body radiotherapy in 4 fractions for primary lung cancer using a stereotactic body frame. *Int J Radiat Oncol Biol Phys.* 2005;63(5):1427-1431.

[4] Fritz P, Kraus HJ, Blaschke T, et al. Stereotactic, high single-dose irradiation of stage I non-small cell lung cancer (NSCLC) using four-dimensional CT scans for treatment planning. *Lung Cancer.* 2008;60(2):193-199.

[5] Baumann P, Nyman J, Hoyer M, et al. Outcome in a prospective phase II trial of medically inoperable stage I non–small-cell lung cancer patients treated with stereotactic body radiotherapy. *J Clin Oncol.* 2009;27(20):3290-3296.

[6] Stephans KL, Djemil T, Reddy CA, et al. A comparison of two stereotactic body radiation fractionation schedules for medically inoperable stage I non-small cell lung cancer: the Cleveland Clinic experience. *J Thorac Oncol.* 2009;4(8):976-982.

[7] Brown WT, Wu X, Fayad F, et al. Application of robotic stereotactic radiotherapy to peripheral stage I non-small cell lung cancer with curative intent. *Clin Oncol (R Coll Radiol).* 2009;21(8):623-631.

[8] Fakiris AJ, McGarry RC, Yiannoutsos CT, et al. Stereotactic body radiation therapy for early-stage non–small-cell lung carcinoma: four-year results of a prospective phase II study. *Int J Radiat Oncol Biol Phys.* 2009;75(3):677-682.

[9] Ricardi U, Filippi AR, Guarneri A, et al. Stereotactic body radiation therapy for early stage non-small cell lung cancer: results of a prospective trial. *Lung Cancer.* 2010;68(1):72-77.

[10] Crabtree TD, Denlinger CE, Meyers BF, et al. Stereotactic body radiation therapy versus surgical resection for stage I non–small cell lung cancer. *J Thorac Cardiovasc Surg.* 2010;140(2):377-386.

[11] Videtic GM, Stephans K, Reddy C, et al. Intensity-modulated radiotherapy–based stereotactic body radiotherapy for medically inoperable early-stage lung cancer: excellent local control. *Int J Radiat Oncol Biol Phys.* 2010;77(2):344-349.

[12] Haasbeek CJ, Lagerwaard FJ, Slotman BJ, Senan S. Outcomes of stereotactic ablative radiotherapy for centrally located early-stage lung cancer. *J Thorac Oncol.* 2011;6(12):2036-2043.

[13] Shibamoto Y, Hashizume C, Baba F, et al. Stereotactic body radiotherapy using a radiobiology-based regimen for stage I nonsmall cell lung cancer: a multicenter study. *Cancer.* 2012;118(8):2078-2084.

[14] Shirata Y, Jingu K, Koto M, et al. Prognostic factors for local control of stage I non-small cell lung cancer in stereotactic radiotherapy: a retrospective analysis. *Radiat Oncol.* 2012;7:1-8.

[15] Inoue T, Katoh N, Onimaru R, et al. Stereotactic body radiotherapy using gated radiotherapy with real-time tumor-tracking for stage I non-small cell lung cancer. *Radiat Oncol.* 2013;8:1-8.

[16] Rwigema JC, Chen AM, Wang PC, et al. Incidental mediastinal dose does not explain low mediastinal node recurrence rates in patients with early-stage NSCLC treated with stereotactic body radiotherapy. *Clin Lung Cancer.* 2014;15(4):287-293.

[17] Shibamoto Y, Hashizume C, Baba F, et al. Stereotactic body radiotherapy using a radiobiology-based regimen for stage I non–small-cell lung cancer: five-year mature results. *J Thorac Oncol.* 2015;10(6):960-964.

[18] Lindberg K, Nyman J, Riesenfeld Källskog V, et al. Long-term results of a prospective phase II trial of medically inoperable stage I NSCLC treated with SBRT – the Nordic experience. *Acta Oncol.* 2015;54(8):1096-1104.

[19] Hayashi S, Tanaka H, Hoshi H. Imaging characteristics of local recurrences after stereotactic body radiation therapy for stage I non-small cell lung cancer: Evaluation of mass-like fibrosis. *Thorac Cancer.* 2015;6(2):186-193.

[20] Hamaji M, Chen F, Matsuo Y, et al. Video-assisted thoracoscopic lobectomy versus stereotactic radiotherapy for stage I lung cancer. *Ann Thorac Surg.* 2015;99(4):1122-1129.

[21] Nagata Y, Hiraoka M, Shibata T, et al. Prospective trial of stereotactic body radiation therapy for both operable and inoperable T1N0M0 non-small cell lung cancer: Japan Clinical Oncology Group Study JCOG0403. *Int J Radiat Oncol Biol Phys.* 2015;93(5):989-996.

[22] Navarro-Martin A, Aso S, Cacicedo J, et al. Phase II trial of SBRT for stage I NSCLC: survival, local control, and lung function at 36 months. *J Thorac Oncol.* 2016;11(7):1101-1111.

[23] Tsurugai Y, Kozuka T, Ishizuka N, Oguchi M. Relationship between the consolidation to maximum tumor diameter ratio and outcomes following stereotactic body radiotherapy for stage I non-small-cell lung cancer. *Lung Cancer.* 2016;92:47-52.

[24] Shaverdian N, Tenn S, Veruttipong D, et al. The significance of PTV dose coverage on cancer control outcomes in early stage non-small cell lung cancer patients treated with highly ablative stereotactic body radiation therapy. *Br J Radiol.* 2016;89(1059):20150963.

[25] Nyman J, Hallqvist A, Lund JÅ, et al. SPACE – a randomized study of SBRT vs conventional fractionated radiotherapy in medically inoperable stage I NSCLC. *Radiother Oncol.* 2016;121(1):1-8.

[26] Miyakawa A, Shibamoto Y, Baba F, et al. Stereotactic body radiotherapy for stage I non-small-cell lung cancer using higher doses for larger tumors: results of the second study. *Radiat Oncol.* 2017;12:1-7.

[27] Swaminath A, Wierzbicki M, Parpia S, et al. Canadian phase III randomized trial of stereotactic body radiotherapy versus conventionally hypofractionated radiotherapy for stage I, medically inoperable non–small-cell lung cancer: rationale and protocol design for the Ontario Clinical Oncology Group (OCOG)-LUSTRE trial. *Clin Lung Cancer.* 2017;18(2):250-254.

[28] Lee S, Song SY, Kim SS, et al. Feasible optimization of stereotactic ablative radiotherapy dose by tumor size for stage I non–small-cell lung cancer. *Clin Lung Cancer.* 2018;19(2):e253-e261.

[29] Cummings MA, Ma SJ, Hermann G, et al. Comparison of single- and five-fraction regimens of stereotactic body radiation therapy for peripheral early-stage non–small-cell lung cancer: a two-institution propensity-matched analysis. *Clin Lung Cancer.* 2018;19(6):511-517.

[30] Karasawa K, Hayakawa S, Machitori Y, et al. Accelerated hypofractionated radiotherapy versus stereotactic body radiotherapy for the treatment of stage I nonsmall cell lung cancer: a single institution experience with long-term follow-up. *Technol Cancer Res Treat.* 2018;17:1533033818806318.

[31] Menoux I, Antoni D, Truntzer P, et al. Stereotactic body radiation therapy for stage I non-small cell lung carcinomas: moderate hypofractionation optimizes outcome. *Lung Cancer.* 2018;126:201-207.

[32] Tsurugai Y, Takeda A, Sanuki N, et al. Stereotactic body radiotherapy for patients with non-small-cell lung cancer using RapidArc delivery and a steep dose gradient: prescription of 60% isodose line of maximum dose fitting to the planning target volume. *J Radiat Res.* 2019;60(3):364-370.

[33] Weiss E, Deng X, Mukhopadhyay N, Jan N. Effects of the recurrence pattern on patient survival following SABR for stage I lung cancer. *Acta Oncol.* 2020;59(4):427-433.

[34] Alongi F, Nicosia L, Figlia V, et al. A multi-institutional analysis of fractionated versus single-fraction stereotactic body radiotherapy (SBRT) in the treatment of primary lung tumors: a comparison between two antipodal fractionations. *Clin Transl Oncol.* 2021;23:2133-2140.
